# Subclinical Carotid Artery Atherosclerosis and Cognitive Function: A Mini-Review

**DOI:** 10.3389/fneur.2021.705043

**Published:** 2021-07-28

**Authors:** Håkon Ihle-Hansen, Hege Ihle-Hansen, Else Charlotte Sandset, Guri Hagberg

**Affiliations:** ^1^Department of Medicine, Bærum Hospital- Vestre Viken Hospital Trust, Drammen, Norway; ^2^Oslo Stroke Unit, Department of Neurology, Oslo University Hospital, Ullevål, Norway

**Keywords:** IMT, cognitive function, dementia, plaque, carotid artery atherosclerosis

## Abstract

Carotid artery atherosclerosis, the result of a multitude of vascular risk factors, is a promising marker for use in risk stratification. Recent evidence suggests that carotid artery atherosclerosis affects cognitive function and is an independent risk factor for the development of cognitive impairment. Both atherosclerosis and cognitive impairment develop over a prolonged period (years), and due to the aging population, markers to identify persons at risk are needed. Carotid artery atherosclerosis can easily be visualized using non-invasive ultrasound, potentially enabling early and intensified risk factor management to preserve cognitive function or delay further decline. However, the burden of atherosclerosis and temporal exposure required to pose a risk of cognitive impairment is unclear. This mini-review aims to explore the available evidence on the association between carotid atherosclerosis and cognition, and furthermore identify the remaining gaps in knowledge.

## Introduction

The prevention of cognitive impairment is one of the most significant challenges of our time with up to 10 million new cases each year (1). The consequences of underlying vascular risk factors such as hypertension increase the risk of both atherosclerosis and cognitive impairment. Monitoring and treating vascular risk factors mid-life is a promising strategy to prevent dementia later in life (2). Furthermore, the burden of atherosclerosis correlates to the underlying burden of vascular risk factors (3) and can be viewed as a surrogate marker of total vascular risk.

Vascular risk factors including atherosclerosis seem to contribute not only to vascular cognitive impairment, but also in the pathophysiology of Alzheimers disease (4). It has been proposed that there may be a convergence of mechanisms in vascular and neurodegenerative processes that cause impairments of cognition. These mechanisms are not yet fully elucidated, but most likely mediated through small vessel disease, endothelial dysfunction, silent ischemia, and reduced cerebral blood flow, which precedes neurodegeneration and amyloid accumulation (5).

Atherosclerosis represents a systemic multifactorial and inflammatory disease affecting the vascular bed (6). It develops gradually over years, first as a thickening of the vessel wall‘s innermost layer - the intima (7), this can either abate or progress into an atherosclerotic plaque and a vascular stenosis (8). Several studies have shown an independent association between atherosclerosis and increased risk of cognitive impairment (9, 10). Predilection sites for atherosclerosis are large and medium-sized arteries as well as areas where laminar blood flow is disturbed.

The carotid artery is ideally placed for ultrasound examination and assessment of atherosclerosis. Different types of angiography examinations are available but either demand more resources or are associated with radiation exposure, leaving ultrasound best suited for screening purposes. Atherosclerosis in the carotid arteries correlates with the presence of atherosclerosis in other vessels (11). Definitions of subclinical atherosclerotic disease in various studies include increased intima-media thickness (IMT), atherosclerotic plaques, and asymptomatic stenosis. Assessment of subclinical carotid artery atherosclerosis holds promise as a marker to identify persons at risk and those who may benefit from intensified risk factor management to prevent further progression of atherosclerosis and cognitive decline.

Symptomatic atherosclerosis in pre- and intra- cerebral vessels increases the risk of brain damage with an associated increased risk of cognitive impairment (12). However, the link between subclinical atherosclerosis (meaning nearly or completely asymptomatic) and cognitive function in stroke-free subjects is not as clear, the available evidence is mainly from large population-based cohort studies.

In this mini-review, focusing on subclinical atherosclerosis located in the carotid arteries, we aim to explore the currently available evidence on the association between carotid atherosclerosis and cognition. Furthermore, evaluate any potential progressive relationships, and identify the remaining gaps in knowledge.

## Subclinical Carotid Atherosclerosis and Cognition

Several large population studies have assessed the association between atherosclerosis and cognitive function, 20,000 with cross sectional design and 50,000 with a longitudinal design with long term follow-up (up 20 years) between initial assessment of atherosclerosis and outcome, i.e., worsening in cognitive performance or dementia (13). The studies included in our mini-review are described in detail in Table 1.

**Table 1:** Excerpts from relevant populations-based studies.

**Author, journal, year study**	**Population size and age**	**Measurements**	**Observation time**	**Outcome**	**Adjusted for**
Gustavsson, Ann Neurol 2020, Malmö Diet and Cancer Study	N 6,103, mean age 57.5	Carotid plaques and IMT	20 years (1991/1994–2014)	*Carotid plaques*: Vascular Dementia HR 1.90 [95% CI 1.07–3.38]. *IMT*: Dementia HR 1.14 [95% CI 1.03–1.26]; Vascular Dementia HR 1.32 [95% CI 1.10–1.57]	Cardiovascular risk factors, education, ApoE, age, sex
Wendel, Stroke 2009, Baltimore Longitudinal Study of Aging	N 538, mean age 54.9	ccIMT	Up to11 years (mean 4 years)	*ccIMT* associated with decline in performance on multiple measures of verbal and nonverbal memory	Age, sex, race, cardiovascular risk factors
Van Oijen, Ann Neurol 2007, Rotterdam Study	N 6,647, mean age 72.4	ccIMT, carotid plaques	mean 9.0 years (1990/1993–1997/1999)	*ccIMT* in the fifth quintile compared with the first, and *Carotid plaques* in 5 or 6 locations compared with subjects without carotid plaques were predictor of dementia	Age and sex and cardiovascular factors
Knopman, Neurology 2001, Atherosclerosis Risk in Communities cohort (ARIC)	*N* 10,963, age range 47–70	ccIMT, divided into tertiles (mean of three sites bilaterally)	Mean 6 years	*ccIMT* were not associated with change in cognitive test scores	Cardiovascular risk factors, sex, race, education level, site, central nervous system medication and age
Wendell, Stroke 2012, Baltimore Longitudinal Study of Aging	N 364, age 60–95 (mean 73.6, median 73)	IMT and carotid plaque	Up to 14 years (mean 6.7, median 7,0)	*IMT:* >2.5-fold increased risk of dementia [HR 2.55 (95% CI 1.32–4.96)] among individuals in the upper quintile of IMT. *Plaque*: Approximately 2.0-fold increased risk of dementia [HR 1.98 (95% CI 1.06–3.70)] among individuals with bilateral plaque.	Cardiovascular risk factors, ApoE
Arntzen, 2012, Cerebrovasc Dis. Tromsø Study	N 4,371	ccIMT and plaque	7 years	*Plaques*: Presence of plaques was significantly associated with change in cognitive test scores. The number of plaques and the total plaque area were associated with lower scores on the verbal memory test. *ccIMT*: No significant association was seen between ccIMT and cognitive test scores.	Sex, age, education, depression and vascular risk factors
Moon, Stroke 2015, Korean Longitudinal Study on Health and Aging	N 348, mean age 71.7	ccIMT and plaque	5 years follow up (2005/2006–20210/2011)	*Plaque*: not associated with cognitive decline after multiple adjustments. *ccIMT*: independent risk factor for the future progression of cognitive dysfunction [HR 1.251 (95% CI 1.006–1.555)].	Hypertension, Cumulative Illness Rating Scale-Geriatric, depression, education and sex.
Johnston, Ann Intern Med 2004, Cardiovascular Health Study	N 4,006 right-handed 65 years of age or older	ccIMT and Left Internal carotid artery stenosis (> or =75% narrowing of diameter)	Up to 5 years	*Left internal carotid stenosis:* Average decrease of more than 1 point annually in Modified Mini-Mental State Examination, OR, 6.7 (95% CI 2.4–18.1) compared with no stenosis. *ccIMT* left side was not associated with cognitive decline after adjustment.	Right-sided stenosis
Romero, Stroke 2009, Framingham study	N1,971 mean age, 58 years	ccIMT and carotid stenosis	Average of 4 years	*Internal carotid stenosis >50%:* associated with poorer performance on executive function (β = −0.42 ± SE 0.18; *P* = 0.02)*ccIMT:* not associated with cognitive function	Age, sex, time to MRI/NP, diabetes, smoking, hypertension treatment, systolic blood pressure, and cardiovascular disease
Sander, Geriatric Psychiatry 2009, INVADE study	N 2,693, mean age 67.7	ccIMT	2 years	*ccIMT:* Significant higher C-IMT in those who developed cognitive decline compared to those who did not. (0.87 vs. 0.78 mm; *p* < 0.0001).	Age, gender, prevalent ischemic heart disease, peripheral artery disease, hypertension, blood glucose, carotid plaques, education level, physical activity and the Geriatric Depression Scale
Newman, JAGS 2005, Cardiovascular Health Study Cohort	N 3,602, median age 74 (65–97)	ccIMT, iIMT and carotid stenosis	Mean 5,4 years	*ccIMT and iIMT:* highest quartile associated with increased risk of dementia [HR1,6 (95% CI 1.1–2.2) vs. 1,5 (95%CI 1.1–2.0) respectively]. *Carotid stenosis:* (regardless degree of stenosis) not associated with dementia	Age, race, education, income, ApoE, and Modified Mini-Mental State Examination score at the time of the brain magnetic resonance scan.
Zhong, Atherosclerosis 2012, Beaver Dam Offspring Study	N 1,651, mean age 66.8	IMT and plaque	Mean 9.2 years (range: 3–13 years)	*IMT:* associated with incidence of cognitive impairment [HR: 1.09, (95% CI: 1.01–1.18)] for each 0.1 mm increase in IMT. *Plaque:* not associated with incident cognitive impairment or cognitive test performance 10 years later.	Age, sex, and education, cardiovascular risk factors and SF-36 mental health
Carcaillon, Alzheimer‘s Dementia 2015, Three-City Study	N 6,025, aged 65–86 years	IMT and plaques	Mean 5.4	*Plaque*: Only plaque were independently related to Vascular or mixed dementia, HR 1.92 [95% CI 1.13–3.22]	Age, sex, ApoE, education, cardiovascular risk factors, personal history of coronary heart disease and stroke
Gardener, 2017, Stroke, Northern Manhattan Study	N 826, mean age 70 years	ccIMT and plaques	Mean 5 years	*ccIMT*: Those with greater ccIMT exhibited worse cognitive performance. *Carotid plaque* not significantly with cognition at baseline or over time.	Age, education, race and vascular risk factors
Komulainen, Neuroepidemiology 2007	*N* 91 women, age 60–70	IMT	12 years	*IMT*: Increased IMT at baseline was an independent predictor for poorer cognitive performance	Age, education, depression, cardiovascular risk factors, cardiovascular disease, hormone replacement therapy, alcohol consumption and physical activity
Hsiu-Fen, Atherosclerosis 2020, Kaohsiung Atherosclerosis Longitudinal Study (KALS)	N 528, mean age 53.9 years	ccIMT and plaques	10 years	ccIMT in the top quartile of and plaques were associated with low 10-year cognitive test scores	Age, sex, educational status, diabetes, hypertension, hypercholesterolemia, and smoking status
Mathiesen, Neurology 2004, Tromsø study	*N* 189 subjects with stenosis was compared to 201 control subjects mean age was 67.7 years	Carotid stenosis (≥35%)	Cross-sectional	*Carotid stenosis:* associated with poorer neuropsychological performance	Age, sex, years of education, MRI lesions, current smoking, and cholesterol-lowering and antihypertensive treatment
Zhong, Atherosclerosis 2011, Beaver Dam	N 2,794, mean age 49 years (21-84)	IMT and plaque	Cross sectional	*IMT* and presence of *plaque* were associated with cognitive performance	Adjusting for age, sex and education
Ihle-Hansen, Journal of Alzheimer's Disease 2019, Cardiac Examination 1950 Study	N 3,413, mean age 63.9 years (63–65)	Carotid plaques	Cross sectional	*Carotid plaque burden* was in contrast to diameter [B –*0.17 (95%* –*0.32* –*0.01)]* or area [B –*0.02 (95%* –*0.03* –*0.01)]* of the thickest plaque not associated with cognitive performance	Sex, education, history of stroke and cardiovascular risk factors
Auperin, Stroke 1996, EVA Study	N 1,389, mean age 65.0 (59–71)	ccIMT and plaques	Cross sectional	*Plaques*: Poor cognitive functioning was associated with plaques. *ccIMT*: there was only a weak association in the subgroup of men with plaques. No association was found in women.	Age, educational level, depressive symptomatology, systolic blood pressure, body mass index, and tobacco and alcohol consumption
Suemoto, Aterosclerosis 2015, ELSA-Brasil	N 8,208, Mean age 49.6	ccIMT	Cross-sectional study	*ccIMT:* associated with worse performance on the delayed word recall (DWRT) [β = −0.433, (95%CI = −0.724; −0.142)].	Age, sex, race, marital status, income, education, cardiovascular risk factors, self-reported heart failure, alcohol use, thyroid function and depression
Zeki Al Hazzouri, Stroke 2015. Coronary Artery Risk Development in Young Adults study	N 2,618, mean age 45.3 years	IMT	Cross sectional. UL at baseline, cognitive test 5 years later	*IMT*: negatively associated with processing speed [−0.06; (95% CI −0.09 to −0.02)]	Age, sex, race, education, glomerular filtration rate and cardiovascular risk factors
Del Brutto, J Stroke Cerebrovasc Dis. 2020, The Atahualpa project	N 561	IMT	cross-sectional	No association	Age and education
Xiang, J Clin Neurosci, 2013	N 2,015	IMT, plaques and stenosis	Cross sectional	*IMT:* OR 1.96 [95% CI 1.23–3.16] and *hyperdense plaque* OR 4.72 [95% CI 2.56–11.2] were associated with poor cognitive performance. Patients with severe (≥70%) *carotid artery stenosis* had a lower Mini-Mental State Examination score compared with the mild to modest (40–70%) carotid artery stenosis group. Cognitive performance differed between patients with left and right carotid artery stenosis.	Age, sex, education, cardiovascular risk factors

*IMT, intima-media thickness; ccIMT, common carotid IMT; iIMT, internal carotid IMT; HR, hazard ratio; CI, confidence interval; OR, odds ratio; VD, vascular dementia; ApoE, Apolipoprotein E*.

Association between subclinical atherosclerosis and cognitive performance in subjects free of known cognitive impairment was explored in 8 studies with a cross sectional design (14–21). Seven cross-sectional studies have found an association between markers of atherosclerosis and cognitive function (14–16, 18–21).

Four studies found an association between greater IMT and reduced performance in some specific cognitive domains (14, 16, 18, 22). The majority of studies included subjects with more advanced atherosclerosis, i.e., plaque, plaque burden, and stenosis (14–16, 20, 21), supporting the notion of an inverse association between increasing atherosclerotic burden and cognitive function.

Nine studies including a total of 23,000 patients showed associations between higher IMT and deterioration of cognitive performance over time (9, 13, 23–30). On the other hand, the level of IMT was not associated with decreased cognitive performance in five studies including in total 27,000 patients (10, 31–34).

The location of the IMT measurement may be of importance. In the Framingham study, greater IMT in the internal carotid artery was associated with impaired cognitive function. However, no such association was found when assessing the common carotid intima-media thickness (ccIMT) and cognitive impairment (32).

Further, eight studies reported associations between carotid plaque/stenosis and decreasing cognitive performance and cognitive impairment in long-time follow-up (10, 13, 27, 30–33, 35), and four studies showed that plaque/stenosis was superior to IMT at predicting cognitive decline (10, 31–33).

## Discussion

There is a significant association between different subclinical atherosclerosis measures and cognition in large population studies, both in studies with cross-sectional design and in longitudinal studies showing progressive changes over time.

The current evidence suggests a stronger association between cognitive impairment and more pronounced subclinical atherosclerosis. In the Cardiovascular Health Study and the Framingham study, asymptomatic ≥50% carotid artery stenosis (conventionally defined as significant atherosclerosis) predicted poorer cognitive performance (10, 32). Both studies failed to find an association between ccIMT and cognition.

Some studies reported no association between continuous measures of IMT and cognitive function, while as a dichotomized variables were significant (35). While increased IMT may represent non-atherosclerotic age-associated changes in the vessel wall, vessel wall tension, or an adaptive response to changes in flow, it is believed that IMT in the upper reference range is less likely to reflect these non-atherosclerotic processes.

Whether the location of atherosclerosis is relevant is still unclear. In the Framingham study, they found that IMT in the internal carotid artery (ICA), in contrast to ccIMT, was more associated with cognitive impairment (32). Atherosclerosis develops earlier in vessel bifurcations and origins such as the carotid bulb and proximal ICA which could explain associations between IMT and cognitive impairment in ICA, but not common carotid artery. However, atherosclerosis (including increased IMT) in other locations than the carotid arteries have also been associated with reduced cognition, supporting the hypothesis that atherosclerosis is a systemic disease of the vascular bed (36).

The exact pathophysiological mechanisms of atherosclerosis-induced cognitive impairment have not yet been identified. Population-based studies do not have the ideal design to illicit an answer and mechanisms may include cerebral changes resulting from silent embolization, inflammation or hypoperfusion (2, 37, 38). Increased arterial stiffness leads to increased pulse-wave velocity,pulsatile pressure and flow in the small vessels (2, 39), and a potential failure in the blood-brain barrier. Since the pathological mechanisms remain unknown, the possibility of reverse causality or that atherosclerosis and cognitive impairment develop in parallel cannot with certainty be excluded. A major limitation of the available evidence is the absence of a universal understanding of how to define and assess subclinical atherosclerosis. Measurement of increased IMT, which is thought to represent the first structural change in the atherosclerotic process, is affected by the exact timing of measurement (varies throughout a cardiac cycle), the location of measurement, and the software algorithm used. Carotid plaques which are more strongly associated with traditional cardiovascular risk factors (3) and proven to be a better predictor of a future cardiovascular event, are highly age-dependent, with a lower prevalence in younger populations (3). Plaque detection rate is also affected by the resolution of the ultrasound devices. As most of the studies were conducted in the ‘90s with older ultrasound devices, an underestimation of plaque occurrence is likely. Furthermore, the definition of plaque is not consistent between the different studies.

As with the assessment of atherosclerosis, evaluation and definition of cognitive impairment lack standardization. The use of different cognitive test batteries, and the definition of cognitive impairment across studies makes it difficult to draw clear conclusions. There are conflicting findings regarding which cognitive domains that are most vulnerable to atherosclerotic carotid disease. In general, vascular cognitive impairment is typically characterized by reduced speed of information processing, complex attention, and frontal-executive functioning (40). However, it seems likely that vascular disease contributes to the cascade of neurodegeneration (2), also affecting other cognitive domains.

## Future Perspectives

Despite associations between atherosclerosis and cognitive function seen in many populations, the amount of atherosclerosis required to pose a risk of cognitive impairment is unclear and is likely both age and person dependent.

Ultrasound of the carotid arteries is a cheap and non-invasive and technique to quantify atherosclerotic burden. Future studies should use standardized imaging protocols, standard definitions of atherosclerosis, and predefined outcome measures. Whether subclinical atherosclerosis poses different risk at different ages and whether different locations, and or intensifying risk factor management can contribute to halting further cognitive impairment needs further exploration.

## Conclusion

Subclinical carotid artery atherosclerosis provides additional information about vascular risk factors burden in realtion to cognitive performance. More research is needed to address whether the assessment of carotid artery atherosclerosis could be used to identify people at increased risk of cognitive impairment and justify intensified risk factor management.

## Author Contributions

All authors listed have made a substantial, direct and intellectual contribution to the work, and approved it for publication.

## Conflict of Interest

The authors declare that the research was conducted in the absence of any commercial or financial relationships that could be construed as a potential conflict of interest.

## Publisher's Note

All claims expressed in this article are solely those of the authors and do not necessarily represent those of their affiliated organizations, or those of the publisher, the editors and the reviewers. Any product that may be evaluated in this article, or claim that may be made by its manufacturer, is not guaranteed or endorsed by the publisher.
